# Aging Neurovascular Unit and Potential Role of DNA Damage and Repair in Combating Vascular and Neurodegenerative Disorders

**DOI:** 10.3389/fnins.2019.00778

**Published:** 2019-08-08

**Authors:** Yan Li, Lv Xie, Tingting Huang, Yueman Zhang, Jie Zhou, Bo Qi, Xin Wang, Zengai Chen, Peiying Li

**Affiliations:** ^1^Department of Anesthesiology, Renji Hospital, School of Medicine, Shanghai Jiao Tong University, Shanghai, China; ^2^Department of Radiology, Renji Hospital, School of Medicine, Shanghai Jiao Tong University, Shanghai, China

**Keywords:** aging, neurovascular unit, cerebral ischemia, DNA damage, DNA repair, blood brain barrier

## Abstract

Progressive neurological deterioration poses enormous burden on the aging population with ischemic stroke and neurodegenerative disease patients, such as Alzheimers’ disease and Parkinson’s disease. The past two decades have witnessed remarkable advances in the research of neurovascular unit dysfunction, which is emerging as an important pathological feature that underlies these neurological disorders. Dysfunction of the unit allows penetration of blood-derived toxic proteins or leukocytes into the brain and contributes to white matter injury, disturbed neurovascular coupling and neuroinflammation, which all eventually lead to cognitive dysfunction. Recent evidences suggest that aging-related oxidative stress, accumulated DNA damage and impaired DNA repair capacities compromises the genome integrity not only in neurons, but also in other cell types of the neurovascular unit, such as endothelial cells, astrocytes and pericytes. Combating DNA damage or enhancing DNA repair capacities in the neurovascular unit represents a promising therapeutic strategy for vascular and neurodegenerative disorders. In this review, we focus on aging related mechanisms that underlie DNA damage and repair in the neurovascular unit and introduce several novel strategies that target the genome integrity in the neurovascular unit to combat the vascular and neurodegenerative disorders in the aging brain.

## Introduction

Aging populations are forcing nations and societies to confront the problem of an increasing prevalence of ischemic stroke and neurodegenerative diseases, including Parkinson’s disease (PD) and Alzheimer’s disease (AD). Specifically, approximately 4.7 million Americans were living with the most common neurodegenerative disease AD in 2010; by 2050 this number reaches 16 million (Hebert et al., 2013). Of note, the epidemiological trends of dementia are expected to cause an immense burden on health-care expenditure (Yang et al., 2013). Meanwhile, our understanding of these neurological disorders is still rudimentary, effective disease-modifying drugs are warranted to treat these disorders.

The neurovascular unit (NVU) is a functional unit with multiple components in the central nervous system (CNS), including vascular cells [pericytes, vascular smooth muscle cells (VSMCs), endothelial cells], glial cells (astrocytes, microglia, oligodendrocytes) and neurons (Banks et al., 2018). These cells together, ultimately determine the function of NVU and their responses in health and disease (Mazeika and Doraiswamy, 2002; Lo and Rosenberg, 2009; Cai et al., 2017). Aging related NVU dysfunction falls into two categories, NVU dysfunction resulting from normal aging or cell senescence and pathological NVU dysfunction under aging-related diseases, such as cerebral ischemic stroke, PD and AD (Cai et al., 2017). The pathological NVU dysfunction has emerged as an important element in the neuronal injury of ischemic stroke and neurodegenerative diseases, in which neuronal cell death, glial cell activation, blood-brain barrier (BBB) disruption, and penetration of peripheral immune cells may ensue with the diseases progress (Mazeika and Doraiswamy, 2002; Lo and Rosenberg, 2009; Cai et al., 2017; Baker and Petersen, 2018). Maintenance of genome integrity is a prerequisite for the maintenance of the normal function of the brain. However, aging not only results in accumulated DNA damages in neurons, the center of the NVU, including DNA breaks, cross-links, and bases mismatch (McKinnon, 2013; Lodato et al., 2018), but also impairs multiple DNA repair mechanisms (Santos et al., 2013; Chow and Herrup, 2015; Zhao et al., 2017). In this review, we provide new insights into DNA damage and repair in neurons and also discuss the potential role of DNA damage and repair in the other cell types of the aging NVU. Novel therapeutic strategies targeting DNA damage and repair that may have the potential to combat the neuronal injury after ischemic stroke and neurodegenerative disorders such as PD and AD will also be introduced.

## Vulnerability of the NVU in the Aging Brain

### Changes of the Aging NVU

**FIGURE 1:**
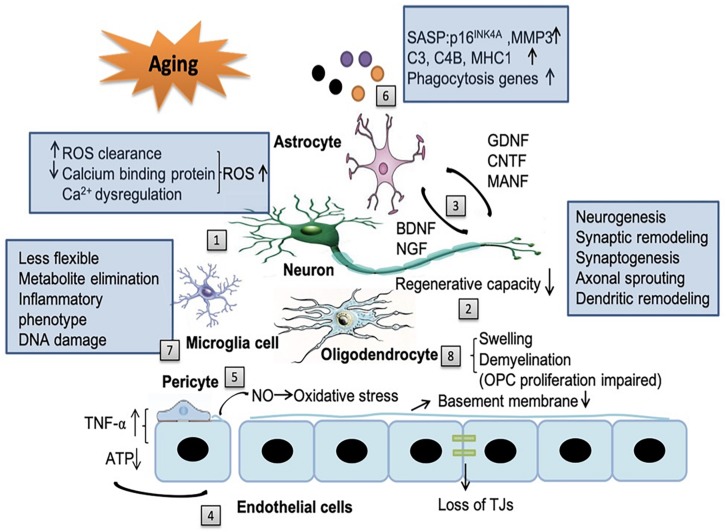
The impact of aging on the neurovascular unit (NVU) components. Aging affects all the components of NVU. [1] In neurons, the capacity for reduced ROS clearance capacity, decreased expression of calcium binding proteins and Ca^2+^ dysregulation induced reactive oxygen species (ROS) production all contribute to the exacerbated neuronal injury. [2] The regenerative capacities of neurons decline in an age-dependent manner, including neurogenesis, synaptic remodeling, synaptogenesis, axonal sprouting and dendritic remodeling. [3] Aging reduces the release of neurotrophic factors from neurons, such as brain-derived neurotrophic factor (BDNF) and nerve growth factor (NGF) and also factors from astrocytes, such as glial cell-line derived neurotrophic factor (GDNF), ciliary neurotrophic factor (CNTF) and mesencephalic astrocyte-derived neurotrophic factor (MANF). The alteration of these factors in aging brain contribute to cognitive decline. [4] With aging, endothelial cells can secrete some noxious factors, such as tumor necrosis factor-α (TNF-α), consume increased amount of ATP, decrease the thickness of basement membrane and disrupt tight junction. In addition, the cross-talk among endothelial cells, astrocytes, and pericytes can modify the secretion of TNF-α. [5] Senile pericytes generate more nitric oxide (NO) and further increase the oxidative stress in the NVU. [6] Senescent astrocytes express senescence-associated secretory phenotype (SASP), such as p16^INK4A^, matrix metallopeptidase 3 (MMP3), complement system genes (C3 and C4B), major histocompatibility complex 1 (MHC1) and upregulate phagocytosis genes, which enhance neuroinflammation and contribute to cognitive decline. [7] With aging, microglia become more rigid and less flexible in mobility. Their capacity to eliminate endogenous metabolites decreases. Aged microglia develop a more inflammatory phenotype and accumulates more DNA damages. [8] Senile oligodendrocytes have swelling morphology and lead to broad and irreversible demyelination which impair the ability of the proliferation of OPCs.

Living cells gradually exhibit features of senescence, which are characterized by sustained cell cycle arrest and release of senescence-associated secretory phenotype (SASP) throughout the body, including the brain (Mazeika and Doraiswamy, 2002; Lo and Rosenberg, 2009; Cai et al., 2017). Compelling evidence indicates that senescence cells in the brain are associated with multiple neurodegenerative diseases (Baker and Petersen, 2018). Meanwhile, dysfunction of NVU in the aging brain plays fundamental role in the pathology of ischemic stroke and also in the progressive neurological deterioration of neurodegenerative diseases (Cai et al., 2017). Since it was first proposed in 2001, the concept of NVU is attracting increasing research attention (Lo and Rosenberg, 2009). With aging, substantial changes occur in all components of the NVU, including neurons, glial cells, vascular cells as well as the basal lamina matrix within the brain vasculature (Mazeika and Doraiswamy, 2002; Lo and Rosenberg, 2009; Cai et al., 2017; Figure 1).

**FIGURE 2:**
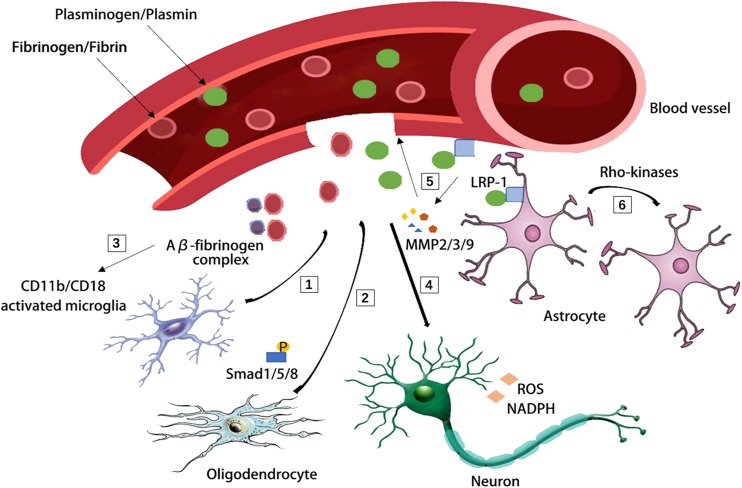
Blood-derived fibrinogen and plasminogen mediated brain pathology through several different mechanisms. [1] Fibrin deposit causes CD11b-CD18 microglia activation in the 5XFAD mouse model of AD. [2] Fibrinogen causes phosphorylation of Smad 1/5/8 and inhibits oligodendrocyte progenitor cells. [3] Aβ-fibrinogen complex enhances chronic CD11b-mediated microglia activation. [4] Fibrin deposit induces reactive oxygen species (ROS) production and triphosphopyridine nucleotide (NADPH) oxidase activation and leads to axonal retraction. [5] The activation of low density lipoprotein receptor-related protein 1 (LRP-1) on endothelial cells was suggested to induce the degradation of tight junctions and base membrane by matrix metallopeptidase-9, matrix metallopeptidase-2, matrix metallopeptidase-3 (MMP-9, MMP-2, and MMP-3). [6] The LRP-1 on astrocytes activates the Rho kinases and mediates the retraction of astrocytic endfeet from vessel walls.

Aging related changes in the NVU usually result in the impaired BBB integrity and penetration of blood derived proteins, such as fibrinogen and plasminogen, which has recently been suggested to clot and form insoluble pro-inflammatory fibrin deposit in the brain (Cortes-Canteli et al., 2015; D’Angelo et al., 2018; Maugeri et al., 2018). Fibrin deposits are associated with amyloid plaques, pericyte loss, dystrophic neurites, and activated microglia in AD, demyelinated multiple sclerosis and stroke (Petersen et al., 2017; Ryu et al., 2018; Brait et al., 2019; Freeze et al., 2019). Fibrin deposits can damage the brain function via a variety of mechanisms: (1) it binds to the CD11b-CD18 integrin receptor and promotes cognitive deficits in a mouse model of AD (Merlini et al., 2019); (2) it can induce reactive oxygen species (ROS) production and the activation of nicotinamide adenine dinucleotide phosphate (NADPH) oxidase, which increases the expression of proinflammatory genes (Ryu et al., 2018; Merlini et al., 2019); (3) it induces adaptive immune responses in the brain and inhibits oligodendrocyte precursor cells (OPCs) and suppresses remyelination (Ryu et al., 2015; Petersen et al., 2017; Lai et al., 2018); (4) it binds Aβ thus forming the Aβ-fibrinogen complex which blocks fibrin degradation and induces neurodegeneration by sustaining fibrin deposition and enhancing chronic CD11b-mediated microglia activation in the CNS (Cortes-Canteli et al., 2010; Zhao et al., 2010; Lai et al., 2018); (5) fibrinogen leads to axonal retraction (Schachtrup et al., 2007) and BBB damage, thus promoting neuroinflammation (Paul et al., 2007). Likewise, plasminogen, the blood protein synthesized in the liver is recently suggested as a regulator of brain inflammatory action and AD pathology (Baker et al., 2018). Depletion of plasminogen/plasmin in peripheral blood, but not in the brain, is highly protective from Aβ deposition and a neuroinflammatory response in a 5XFAD (Tg6799) AD mouse model (Baker et al., 2018; Figure 2).

Aging, with a plethora of mechanisms, affects all the components of the NVU. Aging may also impact the cell-cell interaction within the NVU and ultimately lead to NVU dysfunction and compromised BBB.

#### Aging Neurons in the NVU

In the NVU, neurons are the most susceptible cell type due to their high metabolic rate and are the most vulnerable to aging associated cell injuries (Cai et al., 2017). During aging, their capacity for ROS clearance reduces, making them more vulnerable to oxidative stress (Nicholls and Budd, 2000; Mattson and Magnus, 2006). Meanwhile, calcium dysregulation and decreased expression of calcium binding proteins further increase ROS production, exacerbating neuronal apoptosis and NVU injury in the aging brain (He et al., 1997). On the other hand, the regenerative capacities of neurons decline in an age-dependent manner, including neurogenesis (Smith et al., 2015), synaptic remodeling, synaptogenesis, axonal sprouting and dendritic remodeling (Skaper et al., 2017).

In addition to neuron itself, the interaction between neuron and glia cells may change with aging. There are a variety of neurotrophic factors that have been shown to play important roles in the neuron-glial interaction (Poyhonen et al., 2019). On one hand, the trophic factors produced from neurons, such as brain-derived neurotrophic factor (BDNF) and nerve growth factor (NGF), support the normal function of NVU (Budni et al., 2015). On the other hand, the factors produced from the glial cells, such as glial cell-line derived neurotrophic factor (GDNF), ciliary neurotrophic factor (CNTF), mesencephalic astrocyte-derived neurotrophic factor (MANF) and et al promote synaptic and neuronal growth, pruning, myelination and neuronal cell survival (Poyhonen et al., 2019). However, with aging, the alterations in their expression may contribute to cognitive decline in aging related neurodegenerative diseases (Budni et al., 2015). Astrocytes in the aging brain can also express characteristic SASP proteins, which may turn them into proinflammatory phenotypes and disrupt the functional neuron-glial interaction, thus progressively impair the neuronal functions (Salminen et al., 2011; Chinta et al., 2015).

#### Aging Brain Vasculature

Endothelial cells, one of the key components of the blood brain barrier and the NVU, maintain the integrity of the BBB and the hemostasis of the brain along with the tight junctions between endothelial cells, basement membrane, pericytes and astrocyte endfeet (Lo and Rosenberg, 2009). The cross-talk among endothelial cells, astrocytes, and pericytes of NVU may modify the cytokine secretion patterns of endothelial cells, such as tumor necrosis factor-α (TNF-α) (Banks et al., 2018). With aging, oxidative stress occurs in human endothelial cells and the permeability of BBB increases (Donato et al., 2007; Bake et al., 2009; Lee et al., 2012; Cai et al., 2017). Increased endothelial concentration of TNF-α, decreased thickness of basement membrane, loss of tight junctions (TJs), such as occludin and zonula occludens-1 (ZO-1), reduced secretion of trophic factors from brain pericytes have all been suggested as potential mechanisms underlying the aging associated injury of the NVU (Emerich et al., 2005; Bell et al., 2010; Elahy et al., 2015).

Central nervous system pericytes are embedded in the basement membrane. They generate diverse functional responses with neighboring cells, such as regulating BBB permeability, clearance of toxic metabolites, capillary hemodynamic responses, angiogenesis and neuroinflammation (Sweeney et al., 2016; Villasenor et al., 2017; Khennouf et al., 2018). During aging, the number of pericytes decreases in the brain and the loss of NG2^+^ pericytes is associated with Aβ1-40 in AD patients (Bell et al., 2010). Mechanistically, fibril-enriched preparations (fibril-EP) Aβ1-40 exposure can reduce the cell viability of pericytes and it also increases caspase 3/7 activity, which may induce pericyte apoptosis (Schultz et al., 2018). The PDGF-BB/PDGFRβ (platelet-derived growth factor-BB/platelet-derived growth factor receptor β) signaling plays a pivotal role in the age-dependent pericyte regulation of the BBB integrity. Partially disrupted or deficient of the PDGF-BB/PDGFRβ signaling results in BBB breakdown in an age-dependent manner, which allows accumulation of neurotoxic blood-derived proteins in the brain (Armulik et al., 2010; Sweeney et al., 2016). Senile pericytes contain high smooth muscle actin and the smooth muscle cells generate more nitric oxide (NO), which can react with O^2^-anions, causing severe oxidative stress (Hughes et al., 2006; Cai et al., 2017). Dysfunction of pericytes may ultimately result in comprised blood supply of the NVU due to contractile rigor (Sweeney et al., 2016).

#### The Impact of Aging on Brain Glial Cells

As the most numerous glial cells in the CNS, astrocytes not only provide structural and nutritional supports for neurons, but also participate in the composition of the NVU (Helms et al., 2017; Banks et al., 2018). In senile astrocytes, increased oxidative metabolism and disturbed glutamate regulation can impair their energy support capacity to neurons and cause neuronal excitotoxicity (Maragakis and Rothstein, 2006; Simpson et al., 2010; Cai et al., 2017). Senile astrocytes in the brain also express senescence-associated secretory phenotype (SASP), such as p16^INK4A^ and matrix metalloproteinase 3 (MMP3), which enhances age-related neuroinflammation and exacerbates the functional decline of the aging brain (Salminen et al., 2011; Baker and Petersen, 2018). Recently, the accumulation of senile astrocytes were causally linked to neuronal cell loss and subsequent cognitive impairment (Bussian et al., 2018). Clearance of p16Ink4a-positive senescent astrocytes and microglia using INK-ATTAC transgenic mice prevents gliosis and deposition of neurofibrillary tangle in the cortex and hippocampus (Bussian et al., 2018). On the other hand, aging astrocytes activate the immune system and contribute to cognitive decline. It was recently demonstrated that the complement system genes in astrocytes, such as C3 and C4B were upregulated in the aging brain (Boisvert et al., 2018; Clarke et al., 2018). There’s also an upregulation in antigen presenting genes such as major histocompatibility complex I (MHC I), and phagocytosis genes, such as *Pros1, Mfge8, Megf10, and Lrp1*, thus contributing to the low level inflammatory state characteristic of aging (Mangold et al., 2017; Clarke et al., 2018). However, the MHC I and the phagocytosis gene expression may not be detrimental all the time, they may also promote the clearance of the myelin debris and preserve the cognitive function during aging (Nag and Wadhwa, 2012; Lazarczyk et al., 2016).

Microglia has long been recognized as the main executor of inflammation after acute and chronic brain injuries (Perry and Holmes, 2014; Jin et al., 2017; Kluge et al., 2018). It has been recently shown that neocortical resident microglia are long-lived, with a median lifetime of well over 15 months. About half of the microglia can survive the entire lifespan in mouse (Fuger et al., 2017). With aging, microglia become less flexible in mobility, which makes them become more rigid and less efficient during their daily patrol in the brain (Norden and Godbout, 2013; Perry and Holmes, 2014; Babcock et al., 2015). Their capacity to eliminate endogenous metabolites (e.g. amyloid-beta peptides) also decreases with aging (Norden and Godbout, 2013; Perry and Holmes, 2014; Babcock et al., 2015). Aged microglia develop a more inflammatory phenotype, which is primed, reactive or sensitized, and the activation is amplified and prolonged and more difficult to resolve compared to non-aged microglia (Norden and Godbout, 2013; Perry and Holmes, 2014). In addition, mutations and DNA damages accumulate in microglia with aging (Mrak and Griffin, 2005). Therefore, it’s well recognized that microglial senescence may contribute to age-related neurodegenerative diseases. Although it does not directly participate in the composition of NVU, its interaction with the BBB after central nervous system disorders is getting increasing attention in the field.

Oligodendrocytes are one of the glial cells providing support and insulation to axons in the brain. Senile oligodendrocytes have swelling morphology which includes degenerated myelin sheaths in the cytoplasm (Peters, 2009). With aging, the injuries to myelin and the oligodendrocytes increase while the ability of the brain to repair and renew in response to these injuries declines, leading to broad and irreversible demyelination in various age-related CNS insults (Cai et al., 2017; Petersen et al., 2017). Demyelination results in exposure of unwrapped axons, which in turn stimulates the proliferation of OPCs and remyelination (Petersen et al., 2017). However, the remyelination efficiency is significantly impaired in the aged brain, causing a significantly lower axonal conduction velocity, which may also contribute to the progressive neurological deterioration in those aged individuals with neurodegenerative disorders (Peters and Sethares, 2002; Peters, 2009).

Collectively, the aging related changes in the NVU are illustrated in Figure 1.

### Senile NVU and Cerebral Ischemic Stroke

As the fourth leading cause of death and the primary cause of disability worldwide, stroke poses enormous burden to the family and society (Donahue and Hendrikse, 2018). More than 80% of strokes occur in patients aged 65 or more (Ritzel et al., 2018). Aging is not only a risk factor of cerebral ischemic stroke, but also an indicator of poorer outcome (Cai et al., 2017; Yang and Paschen, 2017). Compared to younger patients, the elderly has higher rates of mortality and disability (Ritzel et al., 2018).

Aging can affect the NVU in a variety of ways (Salinet et al., 2018). Apart from the above mentioned direct impacts of aging on the NVU components, aging can also impair the systemic milieu and alter the immunological response and lead to further exacerbated brain injury after stroke (Pan et al., 2017; Ritzel et al., 2018; Wang et al., 2018). Reduced expression of sirtuin-1 in brain microvessels may also contribute to the increased brain permeability in aged mice after stroke (Stamatovic et al., 2018). However, the capacity of the aged NVU to recover from ischemic stroke is impaired (Jiang et al., 2018). Of note, with aging, BBB breakdown may occur before marked cognitive dysfunction occurs, which may predispose aged patient to post-stroke dementia (Nation et al., 2019). However, the causal link between aging-related NVU dysfunction and post-stroke dementia has not been well-established yet.

### Intimate Association Between AD and NVU Dysfunction

Alzheimer’s disease is a chronic neurodegenerative disease that constitutes 60–70% of dementia cases (Herholz et al., 2018). Emerging evidence suggests BBB disruption may serve as a critical pathway that initiates the onset of AD (Arvanitakis et al., 2016; Sweeney et al., 2018b). Using an advanced dynamic contrast-enhanced magnetic resonance imaging (DCE-MRI) protocol, it’s demonstrated that compromise of the BBB is an early event in the hippocampus during aging and it may contribute to the progressive impairment of cognitive function. Thus, BBB breakdown can also serve as an early biomarker of human cognitive dysfunction (Montagne et al., 2015; Barry Erhardt et al., 2018; Nation et al., 2019).

**FIGURE 3:**
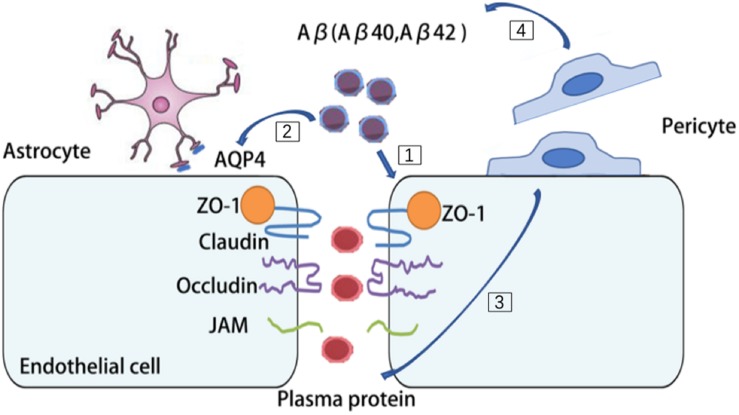
Aβ burden contribute to multiple cell types dysfunction in AD brains. [1] Aβ42 disrupt the organization of tight junctions (TJs) and adherent junction in endothelial cells. [2] Aβ burden redistribute aquaporin 4 (AQP4) on astrocytes and cause retraction and swelling of astrocytic end-feet. [3] In AD brains, pericytes detach from microvessels and cause leakage of plasma protein. [4] Deficiency of pericytes cause accelerated brain Aβ deposition.

Multiple lines of evidence suggest that there are pronounced NVU dysfunction in AD brains (Miners et al., 2018). BBB disruption has been found in the postmortem brain of AD patients, such as substantially reduced TJs proteins, occludin, claudin-5, and ZO-1 in capillaries, leading to increased fibrinogen leakages in the brain parenchyma (Carrano et al., 2011, 2012; Zlokovic, 2011). Aβ42 is likely to disrupt the organization of TJs and adherent junction between endothelial cells, thereby disturbing their barrier function (Carrano et al., 2011, 2012; Zlokovic, 2011). The receptor for advanced glycation end-products (RAGE)-mediated Aβ cytotoxicity is involved in damaging the BBB integrity (Kook et al., 2012; Wan et al., 2015). High degree of Aβ burden is associated with redistribution and even loss of aquaporin 4 (AQP4) on astrocytic end-foot membranes (Yang et al., 2011; Zeppenfeld et al., 2017). Retraction and swelling in astrocytic end-feet is involved in the Aβ mediated impairment of cerebral vascular function and cerebral metabolism (Merlini et al., 2011). Coverages of microvessels by pericytes are significantly reduced in AD brains and it inversely correlated with the extravasation of the blood-drived proteins including immunoglobulin G and fibrin (Sengillo et al., 2013; Winkler et al., 2014). Furthermore, reduced pericyte coverage in AD may further exacerbate cerebral hypoperfusion and Aβ clearance impairment (Dalkara et al., 2011). Deficiency of pericytes causes accelerated brain Aβ deposition and cerebral amyloid angiopathy formation in the brain of a mouse AD model (Sagare et al., 2013). With compelling evidence suggesting an intimate association of NVU dysfunction and AD, it’s reasonable to envision that protecting the aging NVU may be a promising strategy to treat AD (Figure 3).

### Aging NVU and PD

**FIGURE 4:**
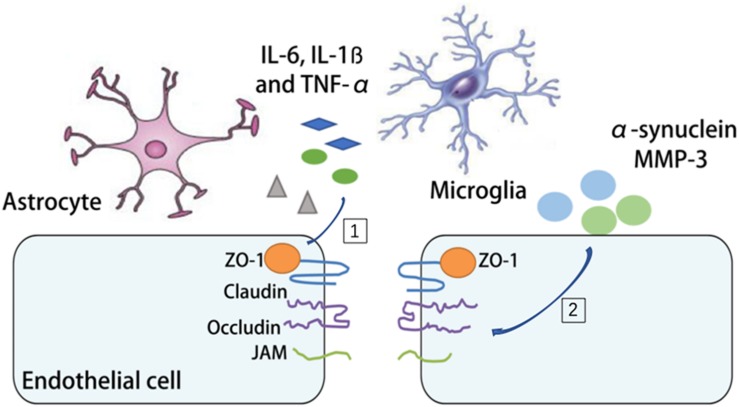
The mechanism of BBB disruption in PD. [1] The release of interleukin 6 (IL-6), interleukin-1ß (IL-1ß) and tumor necrosis factor-α (TNF-α) by microglia and astrocytes during PD is associated with the rearrangement of tight junctions (TJs). [2] The deposition of α-synuclein and matrix metallopeptidase 3 (MMP3) increases BBB permeability and neuroinflammation in PD.

Parkinson’s disease is a progressive long-term neurodegenerative disorder in the aged population. It is characterized by degeneration of dopaminergic neurons in the substantia nigra with accumulated α-synuclein. The relationship between the aging NVU and PD resides in two aspects. On one hand, increased oxidative stress, disturbed mitophagy, reduced energy support and impaired mitochondria repair with aging can all lead to dopaminergic neuronal cell injury and cell loss (Gredilla et al., 2010; Fivenson et al., 2017). On the other hand, the pathological changes of the PD brain, such as α-synuclein deposition and increased MMP-3 alter BBB permeability and neuroinflammation in PD (Chung et al., 2013; Jangula and Murphy, 2013). There is clinical evidence of BBB disruption during PD development (Ohlin et al., 2011; Lee and Pienaar, 2014). By measuring the albumin ratio, it’s demonstrated that PD patients exhibit significant differences in albumin ratio, which indicates BBB dysfunction (Jangula and Murphy, 2013). Several mechanisms have been proposed to explain the BBB disruption in PD. Astrocytes have intimate contact with dopaminergic neurons, especially those with unmyelinated axons (Rodriguez et al., 2014). In the aging brain, instead of providing protection and support, the senile astrocytes along with microglia produce cytokines, chemokines (interleukin 6 (IL-6), interleukin-1ß (IL-1ß) and TNF-α) and ROS, which disrupt the integrity of the NVU, rearrange TJs ZO-1 and occludin and propel disease progression (Gredilla et al., 2010; Collins et al., 2012; Rodriguez et al., 2015; Fivenson et al., 2017). In addition, increased oxidative stress, neuroinflammatory processes and infiltration of peripheral immune cells have all been linked to BBB disruption in PD, although still controversial (Block et al., 2007; Appel, 2009; Chung et al., 2016). So far it still remains unknown whether NVU targeted therapy could be protective against PD (Figure 4).

## Accumulated DNA Damage in Multiple Cell Types in the Aging NVU

Progressive DNA damage accumulation is linked to cellular senescence and organismal aging. Most DNA damage is referred to oxidative DNA damage, an endonuclease-independent process resulting from ROS attacks, posing a major threat for the genome stability in the aging brain (McKinnon, 2013). There are several types of DNA damage, such as DNA breaks, cross-links, and bases mismatch (e.g., oxidative lesions) (Hayflick, 2007), among which DNA strand breaks are the most frequently encountered and detrimental. When classified depending on their mechanisms of action, DNA damage can be divided into two distinctive types: active DNA damage and passive DNA damage. It’s emerging that DNA damage accumulation underlies age-related neurodegenerative diseases, such as stroke, AD and PD [88]. Among the NVU cells, neurons are the most widely studied cell type in terms of DNA damage and repair. However, DNA damage in endothelial cells and glial cells are also getting increasing attention due to their fundamental role in the NVU. We summarize different types of DNA damages in different neuronal cells in the NVU following ischemic stroke, AD and PD in Supplementary Table 1.

### Accumulated DNA Damage in the NVU and the Relationship With Neurodegenerative Diseases

Neurons are highly vulnerable to oxidative stress induced DNA damage due to its high metabolic and mitochondrial activity, oxygen consumption, as well as high rates of transcription and translation (Lu et al., 2014; D’apolito et al., 2018). This condition, together with the impaired DNA repair capacity, can lead to a high accumulation of DNA damage which ultimately result in neurodegenerative processes (Choi et al., 2012; Suberbielle et al., 2015; Hou et al., 2018). Persistent accumulation of DNA damages, including oxidative DNA damages, single-strand breaks (SSBs), and double-strand breaks (DSBs), have been found in neurons of both AD and PD patients and experimental animal models (Adamec et al., 1999; Abolhassani et al., 2017). Cortical neurons prepared from 8-oxodeoxyguanine/8-oxoguanine glycosylase (8-oxo-dG/OGG1) double deficient adult mouse brains exhibit increased 8-oxoguanine (8-oxoG) accumulation in mitochondrial DNA and significantly poor neuritogenesis *in vitro* in the absence of antioxidants in AD (Abolhassani et al., 2017). In PD patients, a significant increase in 8-hydroxy-2’-deoxyguanosine (8-OHdG) levels was shown in mtDNA in nigrostriatal dopaminergic neurons [69]. After ischemic stroke, there are also pronounced formation of apurinic/apyrimidinic (AP) sites base modifications (e.g., 8-oxodG, 8-OHdG, and 8-oxoG), SSBs and DSBs in ischemic neurons (D’apolito et al., 2018). The DNA damages in neurons of different neurodegenerative diseases are summarized in Supplementary Table 1.

DNA damages also surface in brain vascular endothelial cells and lead to endothelial apoptosis and cell loss, which contributes to cognitive decline in AD and ischemic stroke via a number of mechanisms (Chen et al., 1997; Ou and Schumacher, 2018): (1) increased apoptosis in endothelial cells results in reduced cerebral blood flow due to stiffened vasculature; (2) compromised BBB integrity can cause unregulated passage of blood-derived protein into the brain and increased infiltration of peripheral immune cells; (3) generalized NVU dysfunction, which will impact both astrocytes and neurons, leads to reduced function of the NVU and a pro-inflammatory environment in the brain (Jadavji et al., 2015). Detailed DNA damage types identified in endothelial cells are also summarized in Supplementary Table 1.

Astrocytes play critical roles in the maintenance and protection of neurons especially acute or chronic injury encountered, they are also vulnerable to oxidative DNA damage (Fluteau et al., 2015). Astrocyte oxidative DNA damage and their response to oxidative DNA damage are related to the progression of cognitive impairment (Waller et al., 2018) and cerebral ischemic injuries (Chen et al., 1997; Matsuda et al., 2009). In addition, the activation of astrocytes has a profound impact on the glial scar formation and NVU remodeling after stroke (Nahirney et al., 2016; Yanev et al., 2017; Blochet et al., 2018). However, evidence of DNA damages in astrocytes in AD or PD is relatively limited. There is even conflicting data showing reduced DNA-dependent protein kinase expression and DNA-damage associated molecules H2A histone family member X (H2AX) in Alzheimer’s disease (Simpson et al., 2010). Therefore, the role of astrocytic DNA damage in the development of neurodegenerative diseases remains obscure and warrants further investigation.

Pericytes are adjacent to capillaries and essential to the regulation and limitation of the transport of various substances, thereby play important roles in the regulation of the BBB permeability and NVU functions (Bell et al., 2010; Fernandez-Klett et al., 2013; Montagne et al., 2018; Sweeney et al., 2018a). The oxidative DNA damage and repair in pericytes are poorly understood comparing to neuronal cells and endothelia cells. However, pericyte loss has been evidenced as an important contributor for BBB disruption and NVU dysfunction in AD, white matter dysfunction and ischemic stroke (Bell et al., 2010; Fernandez-Klett et al., 2013; Montagne et al., 2018; Sweeney et al., 2018a). Pericytes incubated with Aβ express high levels of cyclin D1 and integrin alpha 5 (ITGa5), which represents disturbed cell cycle and increased cell apoptosis (Davis et al., 1999; Miao et al., 2005). Thymosin β10 (Thyβ10) is supposed to mediate the Aβ induced compromise of the actin network and increased expression of cyclin D1 and Thyβ10 in AD brain, may eventually lead to apoptosis in cerebrovascular cells (Gugliandolo et al., 2018; Knopman, 2019). However, whether different types of DNA damages participate in the pericyte loss during neurodegenerative diseases remains to be explored.

Collectively, there are compelling evidence showing that a variety of DNA damage molecules in neurons contribute to ischemic stroke, AD and PD, such as 8-OHdG, 8-oxodG, γ-H2AX and et al. as listed in Supplementary Table 1. While oxidative DNA damage and repair in other cell types of the NVU remains poorly understood and warrants further investigation.

### Impaired DNA Damage Responses in the NVU in the Aging Brain

Once DNA damage occurs, it can be sensed by DNA damage responses (DDR), several signaling pathways can be triggered to initiate cell injury (Lee and Paull, 2005; Ciccia and Elledge, 2010; Shiloh, 2014; Lavin et al., 2015). Phosphoinositide 3-kinase (PI3K)-related kinases (PIKKs) can phosphorylate downstream proteins to activate DNA damage responses. There are three types of PIKKs DNA-dependent protein kinase that can be recruited to DNA damage sites, DNA-dependent protein kinase catalytic subunit (DNA-PKcs), Ataxia-telangiectasia mutated (ATM), and taxia-telangiectasia and Rad-3-related (ATR) (Blackford and Jackson, 2017). DNA-PKcs is required to form an active DNA-PK complex with Ku70/80 for DNA repair of double strand breaks, which is accompanied by non-homologous end-jointing (NHEJ), which will be discussed later. ATR is activated by single strand break and stalled DNA replication forks while ATM can be activated directly by oxidative stress and DNA damage, mainly double strand DNA damage (Li et al., 2011; Blackford and Jackson, 2017; D’apolito et al., 2018). Once ATM is activated, it phosphorylates a multitude of substrates, including nuclear respiratory factor 1 (NRF1), p53-binding protein 1 (53BP1), mediator of DNA damage checkpoint protein 1 (MDC1), breast cancer type 1 susceptibility protein (BRCA1) MRE11/RAD50/NBS1(MRN) and H2AX to activate cell cycle checkpoints and initiate DNA repair. When ATM phosphonates NRF1, it leads to enhanced electron transport chain and mitochondrial function (Chow et al., 2019). If the damage is too severe to be repaired, ATM can direct the cell to apoptosis (Lee and Paull, 2005; Ciccia and Elledge, 2010; Shiloh, 2014; Lavin et al., 2015).

**FIGURE 5:**
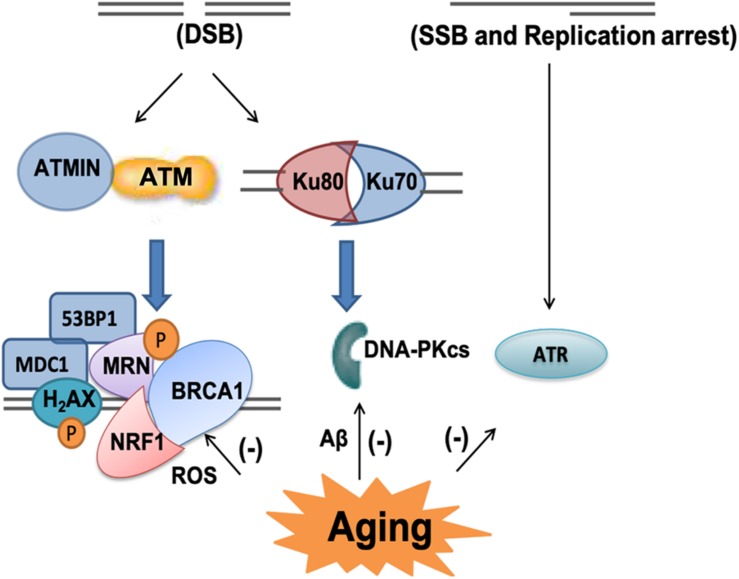
PIKKs-related signaling pathway in the aging brain. There are three types of phosphoinositide 3-kinase (PI3K)-related kinases (PIKKs) DNA-dependent protein kinase including ataxia-telangiectasia mutated (ATM), DNA protein kinase catalytic subunit (DNA-PKcs) and taxia-telangiectasia and Rad-3-related (ATR). ATM can be activated by double-strand breaks (DSB) and it phosphorylates a multitude of substrates, including nuclear respiratory factor 1 (NRF1), H2A histone family member X (H2AX), mediator of DNA damage checkpoint protein 1 (MDC1), p53-binding protein 1 (53BP1), breast cancer type 1 susceptibility protein (BRCA1) and MRE11/RAD50/NBS1 (MRN). As an interactor of ATM, ATMIN plays important role in its response to oxidative stress and aging related DNA damage. DNA-PKcs is required to form an active DNA-PK complex with Ku70/80. ATR is activated by single-strand breaks (SSB) and replication arrest. In aging and AD brain, all these DNA damage response pathways have been shown to progressively decline.

Among the three PIKKs, both ATM and DNA-PKcs have been implicated to play fundamental roles in aging and neurodegenerative diseases (Chung, 2018; Gal et al., 2018). DNA-PKcs has been found in the amygdala of cognitively impaired patients (Gal et al., 2018). Both Aβ (1-42) oligomers and aggregated Aβ (25-35) have been shown to inhibit DNA-PK kinase activity, compromising double strand break repair (NHEJ) and sensitizing cells to non-lethal oxidative injury (Cardinale et al., 2012). ATM-centered DNA damage response has been shown to progressively decline with aging, but significantly increased phospho-ATM immunoreactivity has been found in dopaminergic neurons in PD mouse model (Cornelis et al., 2018; Milanese et al., 2018; Qian et al., 2018). Excessive ROS stimulates ATM-dependent phosphorylation of drebrin, a regulator of cytoskeletal functions during neuronal development. The phosphorylation of drebrin improves structural and functional changes in the aging and AD synapse by enhancing the protein stability (Kreis et al., 2019). Depletion of ATM interactor (ATMIN), an interactor of ATM in the murine nervous system resulted in reduced numbers in dopaminergic neurons and increased mortality of aging mutant mice (Kanu and Behrens, 2008; Kanu et al., 2010; Figure 5).

**FIGURE 6:**
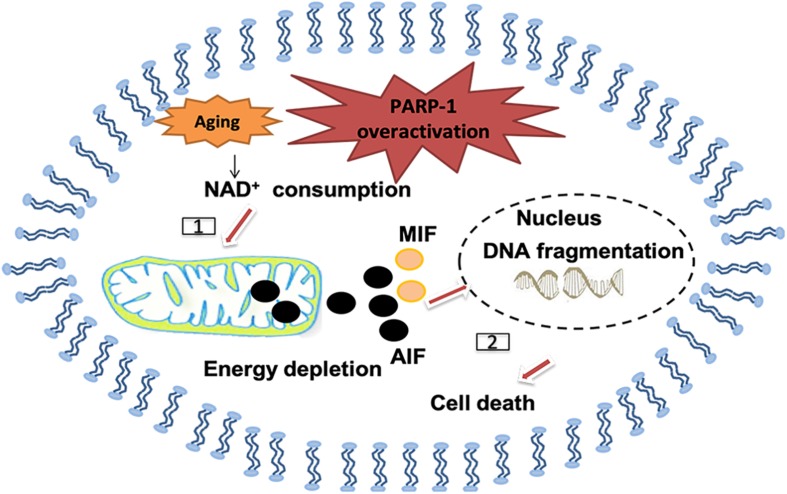
Polymerase-1 over-activation causes cell death in the senile brain. [1] NAD^+^ as the substrate for poly ADP-ribose polymerase (PARP) may be depleted and causes energy depletion in mitochondrial, especially in the senile brain. [2] PAR may directly cause the release of apoptosis inducing factor (AIF) from mitochondrial and transport into the nucleus, thus promoting macrophage migration inhibitory factor (MIF) to translocate from the cytoplasm to the nucleus and cause DNA fragmentation.

Poly (ADP-ribose; PAR) polymerase-1 (PARP-1) is another enzyme that can be activated by oxidative stress and DNA damages, including single and double strand breaks. It belongs to NAD^+^-dependent enzymes in eukaryotes. Cellular DNA damages, especially SSB, trigger overactivation of PARP-1, followed by NAD^+^ depletion and lead to depletion of energy supply and mitochondrial impairment (Vosler et al., 2009; Fang et al., 2014). PARP-1 activation helps translocation of apoptosis-inducing factor (AIF) from the mitochondrion to the nucleus and also for the macrophage inhibitory factor (MIF) to translocate from the cytoplasm to the nucleus, where both of them can induce pro-death processes (Wang et al., 2016; D’apolito et al., 2018). Importantly, PARP-1 is not always detrimental, although overactivation of PARP-1 usually leads to pro-death cellular signals, its recognition of DNA damage is also important in initiating DNA repair (Vida et al., 2016). Of note, significant changes in the activity of PARPs have been detected in ischemic stroke (Andrabi et al., 2011; Nakajima et al., 2015), AD (Martire et al., 2016) and PD (Kam et al., 2018; Olsen and Feany, 2019; Figure 6).

As described above, mounting evidence suggests that aging not only results in accumulated DNA damages in the NVU comprising cells, but also affects the DNA damage response pathways, which contribute to the evolution of neuronal injuries in ischemic stroke and also neurodegenerative diseases. On the other hand, cells are equipped with delicate mechanisms to repair the DNA damages. However, with aging, the DNA repair capacities are impaired, which are associated with exacerbated ischemic brain injury and the progression of neurodegenerative diseases as we will discuss in the following part.

### Impaired DNA Repair Capacities in Aging Brain

**FIGURE 7:**
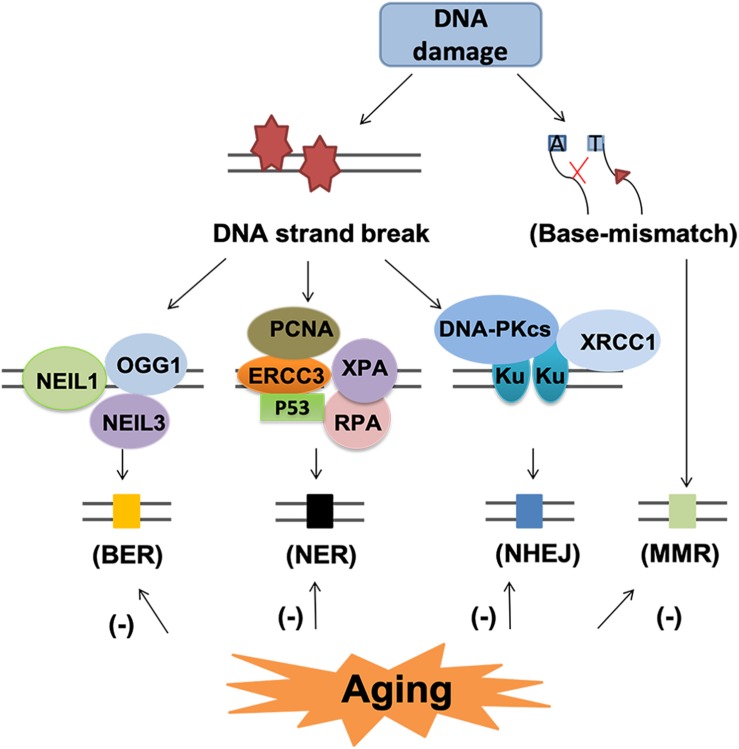
DNA repair pathway and subsequent events in the aging brain. DNA repair pathway include base excision repair (BER), nucleotide excision repair (NER), non-homologous end-joining (NHEJ) and mismatch repair (MMR). Each pathway involved several various molecules. BER and NER are basic nuclear and mitochondrial DNA repair pathway for DNA strand break. NHEJ and MMR are two different pathways for the repair of base-mismatch. All the repair mechanisms become less effective with age, thus, may lead to accumulated DNA lesions and increase the risk of neurodegenerative diseases.

Although DNA damage is inevitable during aging, the cells have endogenous mechanism to repair these damages. The main DNA repair pathways include base excision repair (BER), nucleotide excision repair (NER), homologous recombination (HR), NHEJ and DNA mismatch repair (MMR) (Wyman and Kanaar, 2006; Iyama and Wilson, 2013; Figure 7).

Base excision repair is the primary nuclear and mitochondrial repair pattern for oxidative DNA damage, recognizing and replacing DNA modifications characterized by small base modifications (Li et al., 2011, D’apolito et al., 2018). BER is initiated by various DNA glycosylases, such as OGG1, endonuclease III-like 1 (NTH1), endonuclease VIII-like 1 (NEIL1) and endonuclease VIII-like 3 (NEIL3). These glycosylases excise the damaged bases and generate an AP site in DNA. The resulting AP site is then recognized by AP endonuclease 1 (APE1), which cleaves the phosphodiester backbone 5’ to the AP site, leaving a nick with a 3’-OH group and a 5’-deoxyribose phosphate (dRP) residue. Next, the dRP is removed and filled in by “short-patch” repair or “long-patch” repair (D’apolito et al., 2018). Short patch repair involves sequential single-nucleotide gap-filling in which DNA polymerase beta (Polβ) synthesizes a single nucleotide to fill the gap and DNA ligase III-XRCC1 complex seals the nick between the newly synthesized nucleotide and the DNA template. Long patch repair is initiated by flap endonuclease-1 (FEN1) and usually produces a repair tract of at least two, most frequently four nucleotides (D’apolito et al., 2018).

With aging, the BER process becomes less effective (Niedernhofer et al., 2018), leading to accumulated DNA damage lesions that contribute to age-related degenerative diseases (Santos et al., 2013; Chow and Herrup, 2015). Firstly, the reduction in DNA damage recognition by reduced expression of DNA-PK and H2AX leads to impaired DNA damage recognition, which is associated with neuronal cell loss and glial responses in AD (Myung et al., 2008; Simpson et al., 2010). Secondly, the declined glycosylase activity and reduced DNA polymerase activity have been linked to aging related reduction of BER (Best, 2009). In the aged cerebellum, uracil DNA glycosylase (UDG) declined nearly 50% and OGG1 declined 90% (Best, 2009). In human studies, impaired BER function and the decreased BER capacity could be related to a reduction of Polβ activity and decreased glycosylase UDG in aging related neurodegenerative diseases (Sykora et al., 2013; Leandro et al., 2015). Recently, epigenetic events such as DNA methylation have also been identified to contribute to aging process and defective BER repair system (Dor and Cedar, 2018). Hypermethylation of the BER-related genes results in decreased expression these genes, especially OGG1, which is associated with reduced BER-related incision activity in AD and PD (Blanch et al., 2016).

The NER pathway is a versatile and complex system able to repair different types of DNA helix distorting lesions. In the aged humans, there are reduced expression of NER proteins, such as complementation group 3 (ERCC3), proliferating cell nuclear antigen (PCNA), replication protein A (RPA), xeroderma pigmentosum A (XPA) and tumor protein p53 (p53) (Gorbunova et al., 2007). However, the mechanism underlying this decline remains obscure. Treatment with short oligonucleotides that mimics DNA damage upregulated the levels of NER proteins in aged cells, suggesting decreased NER enzymes or altered induction of DNA damage response may underlie the decline of NER activity (Goukassian et al., 2002). The expression of NER proteins, such as ERCC1, is suggested to be associated with AD and PD (Sepe et al., 2016; Jensen et al., 2018).

Non-homologous end-jointing is a process to restore double stranded breaks of DNA. DNA-dependent protein kinase (DNA-PK) and its activator Ku play important roles initiating the NHEJ process upon binding to the DNA ends of DSBs (Kanungo, 2016). Reduced NHEJ activity as well as DNA-PKcs and Ku protein levels have been shown in aging and AD brains (Vyjayanti and Rao, 2006; Sharma, 2007; Kanungo, 2016). The level of Ku may be one of the mechanisms underlying the age-related decline of NHEJ (Rulten and Caldecott, 2013). Deficiency of Ku80 can induce Aβ generation, which in turn potentiates the degradation of DNA-PKcs and consequently reduced activity of NHEJ (Rulten and Caldecott, 2013).

Mismatch repair system provides two main genetic stabilization functions: correction of errors generated during replication and ensuring the fidelity of recombination. Mutations in MMR genes increase with aging but the function of MMR declines (Bak et al., 2014). The mutagenic MMR has detrimental effects in neurodegenerative diseases for repeat expansions promotion (Bak et al., 2014). For AD patients, elevated DSB accumulation, reduction of DSB repair proteins such as DNA-PKcs and MRN complex proteins, and impaired BER activity all contribute to cell apoptosis or permanent cell-cycle arrest (Andreassi, 2008; Madabhushi et al., 2014).

Therefore, both increased DNA damage accumulation and defected DNA repair mechanisms have been identified in ischemic stroke, AD and PD. However, most of the above-mentioned studies focus the changes in neurons, but not other cell types of the NVU. Thus, it still remains unknown whether the above changes are directly associated with the NVU dysfunction in the aged brain. But, we envision that DNA damage and repair targeted therapy may help to preserve the NVU function and thus improve the outcome of these diseases. In the following part, we will introduce some potential therapeutic strategies that target DNA damage and repair in the treatment of ischemic stroke and neurodegenerative disorders.

## Potential Therapeutic Strategies Targeting DNA Damage and Repair in Age-Related Vascular and Neurodegenerative Disorders

Several strategies have been proposed to combat the DNA damage in neurodegenerative diseases, such as prophylactically or therapeutically using antioxidants. Glutathione (GSH), vitamin E, selenium and superoxide dismutase have all been suggested to protect against oxidation and reduce DNA damage directly or stimulate the activity of DNA repair enzymes (Tomasetti et al., 2001; Mangialasche et al., 2010; Persson et al., 2014; Poprac et al., 2017).

Stimulating nuclear factors or increasing the activity of DNA repair enzymes can be another potential strategy to reduce the DNA damage and combat the neurodegenerative diseases. Forkhead box O3 (FOXO3a), the main isoform of FOXO transcription factors, has been shown to regulate the DNA repair genes. Inhibition of FOXO3a is associated with suppressed DNA damage response and attenuated Alzheimer-type pathology (Fluteau et al., 2015; Shi et al., 2016). In addition, there are a variety of DNA repair enzymes and proteins that can be targeted to combat the neuronal injuries, such as Apurinic/apyrimidinic endonuclease 1 (APE1), ATMIN, endonuclease VIII-like1 (NEIL1) DNA glycosylase, Polβ and DNA polymerase gamma (Polγ). APE1 is a critical BER enzyme that repairs AP sites. It has been shown to enhance neuronal survival after oxidative stress, provide neuroprotection against ischemic brain injury (Stetler et al., 2010; Leak et al., 2015) and also protect against Aβ25-35-induced neurotoxicity (Kaur et al., 2015). Administration of an endogenous small neuropeptide, pituitary adenylate cyclase-activating polypeptide (PACAP), is shown to induce APE1 expression and thus reduces oxidative DNA stress and reduce neuronal cell death after cerebral ischemia (Stetler et al., 2010). In addition, direct NAD repletion could markedly restore the DNA repair activity and reduce the accumulation of DNA damage (Wang et al., 2008). ATMIN is an essential component of the ATM signaling pathway (Kanu et al., 2010). ATMIN has same function in DNA repair system and deficiency of ATMIN will cause neurological disorders (Kanu and Behrens, 2008). Endonuclease VIII-like1 (NEIL1) DNA glycosylase is one of eleven mammalian DNA glycosylases that take part in the first step of the BER pathway (Prakash et al., 2016). Early research showed that NEIL1 function is important in brain-related diseases, such as AD (Canugovi et al., 2014). Polβ renders neurons vulnerable to reductions in cellular energy levels, sustain or elevate Polβ or DNA repair levels may protect neurons against dysfunction and degeneration in aging and AD (Sykora et al., 2015). Suitable supplement of Polγ may relieve PD syndrome (Gaweda-Walerych and Zekanowski, 2013). Collectively, these prophylactic or therapeutic DNA damage and repair targeting treatments appear to be a promising strategy to combat the DNA damage in neurodegenerative diseases Supplementary Table 2.

## Conclusion

Aging related accumulation of DNA damage is emerging as important mechanism that underlies the neuronal injuries after ischemic stroke and neurodegenerative diseases like AD and PD. On the other hand, recent studies have highlighted the important role of NVU dysfunction and subsequent BBB damage and penetration of blood-derived substances, such as fibrinogen and plasminogen in the development of these diseases. In addition, aging related changes in the DNA damage responses and DNA repair signaling are also intimately associated with the progression of different types of age-related neurodegenerative diseases. Therefore, we envision that targeting DNA damage and DNA repair may serve as a promising strategy to combat the NVU dysfunction after ischemic stroke and neurodegenerative diseases. Although a variety of DNA damage and repair targeted treatments have been proposed to treat these neurological disorders, it still remains unknown whether these treatments can effectively improve the NVU function in these neurological disorders. Further investigations are warranted for a better understanding of DNA damage and repair related NVU dysfunction to explore new treatment ideas for multiple neurodegenerative disorders.

## Author Contributions

All authors listed have made a substantial, direct and intellectual contribution to the work, and approved it for publication.

## Conflict of Interest Statement

The authors declare that the research was conducted in the absence of any commercial or financial relationships that could be construed as a potential conflict of interest.
