# Supplementary material for: Aging Neurovascular Unit and Potential Role of DNA Damage and Repair in Combating Vascular and Neurodegenerative Disorders
Source: Front Neurosci. 2019 Aug 8;13:778. doi: 10.3389/fnins.2019.00778 (PMC6694749; doi:10.3389/fnins.2019.00778)
Supplement: Supplementary file 1 [file Table_1.DOCX]

**Table 1. DNA damages in different cell types of NVU in neurodegenerative diseases**

| DNA damage molecule | Mechanisms of action | Cell type | Reference |
| --- | --- | --- | --- |
| AD | | | |
| 8-OHdG | A hallmark of oxidative DNA damage | Neuron | (Brasnjevic et al., 2008) |
| 8-oxodG | Lead to GC-TA transversions | Neuron | (Lee and Pfeifer, 2008;Abolhassani et al., 2017) |
| γ-H2AX | Decrease BER activity | Neuron,glial cells | (Simpson et al., 2010a) |
| 8-oxoG | Lead to poor neuritogenesis | Neuron | (Sliwinska et al., 2016;Banda et al., 2017) |
| DNA-PKcs  OGG1 | Decrease BER activity  Reduce BER-related incision activity | Neuron,glial cells  Neuron | (Simpson et al., 2010a;Madabhushi et al., 2014)  (Sliwinska et al., 2016;Coppede et al., 2017) |
| PD |  |  |  |
| 8-OHdG | A hallmark of oxidative DNA damage | Neuron | (Chung et al., 2016) |
| 8-oxodG | Lead to GC-TA transversions | Neuron | (Lee and Pfeifer, 2008) |
| 8-oxoG | Lead to poor neuritogenesis | Neuron | (Banda et al., 2017) |
| OGG1 | Reduce BER-related incision activity | Neuron | (Dor and Cedar, 2018) |
| Stroke |  |  |  |
| 8-OHdG | A hallmark of oxidative DNA damage | Neuron, astrocyte, microglia | (Matsuda et al., 2009;Li et al., 2011;Li et al., 2018) |
| 8-oxodG | Lead to GC-TA transversions | Neuron | (Lee and Pfeifer, 2008;Li et al., 2011;Li et al., 2018) |
| 8-oxoG | Lead to GC-TA transversions | Neuron | (Li et al., 2011;Banda et al., 2017;Li et al., 2018) |
| DNA-PKcs | Decrease BER activity | Neuron | (Matsuda et al., 2009;Li et al., 2011) |
| OGG1 | Reduce BER-related incision activity | Neuron | (Li et al., 2011;Li et al., 2018) |

NVU, Neurovascular unit; AD, Alzheimers’ disease; PD, Parkinson’s disease; BER, Base-excision repair; 8-OHdG, 8-hydroxy-2’-deoxyguanosine; 8-oxodG, 8-oxodeoxyguanine; γ-H2AX, Gamma-H2A histone family member X; 8-oxoG, 8-oxoguanine; DNA-PKcs, DNA protein kinase catalytic subunit; OGG1, 8-oxoguanine glycosylase.

**Table 2. Therapeutic strategies in combat DNA damage accumulation the aging related neurodegenerative diseases.**

| Treatments | Mechanisms of action and therapeutic target | Disease | Reference |
| --- | --- | --- | --- |
|  |  |  |  |
| GSH | Antioxidant that react with ROS and oxidized products forming glutathione disulphide (GSSG) | AD | (Persson et al., 2014;Poprac et al., 2017) |
| Vitamin E | Endogenous antioxidants that affect the phagocytosis process of Aβ and protect against lipid peroxidation | AD | (Mangialasche et al., 2010;Persson et al., 2014) |
| Selenium | Functions as a cofactor in important antioxidant enzymes such as TrxR. | AD | (Loef et al., 2011;Persson et al., 2014) |
| Superoxide dismutase | Defend against ROS and catalyse the conversion of O_2_ ∙− to H_2_O_2_ and oxygen | AD | (Persson et al., 2014) |
| FOXO3a | Triger cellular responses to oxidative stress and mediates adaptive responses | AD | (Vogt et al., 2005;Fluteau et al., 2015) |
| APE1 | Repairs AP sites by cleaving the phosphodiester backbone 5’ to the AP site | AD and stroke | (Stetler et al., 2010;Leak et al., 2015;Dumitrache et al., 2018) |
| ATMIN | Participate in DNA repair system, especially for DSBs repair. | PD | (Kanu and Behrens, 2008) |
| NEIL1 | recognizes and cleaves mainly oxidized pyrimidines and hydantoin lesions from DNA and remove DNA lesion during nucleotide excision repair | AD | (Jaruga et al., 2010;Zhao et al., 2010) |
| Polβ | Repair oxidative DNA damage | AD | (Wei and Englander, 2008;Sykora et al., 2013;Sykora et al., 2015) |
| Polγ | Possess a 3' to 5' exonuclease activity which important in the repair process | PD | (Luoma et al., 2007;Anvret et al., 2010;Gaweda-Walerych and Zekanowski, 2013) |

AD, Alzheimers’ disease; PD, Parkinson’s disease; GSH, Glutathione; ROS, Reactive oxygen species; TrxR, Thioredoxin reductase; FOXO3a, Forkhead box O3; APE1, Apurinic/apyrimidinic endonuclease 1; AP site, Apurinic/apyrimidinic site; DSBs, Double-strand breaks; ATMIN, ATM interactor; NEIL1, Endonuclease VIII-like1; Polβ, DNA polymerase beta; Polγ, DNA polymerase gamma.
